# Large Peripheral Osteoma of the Mandible: A Case Report

**DOI:** 10.1155/2010/834761

**Published:** 2011-02-06

**Authors:** Emel Bulut, Aydan Acikgoz, Bora Ozan, Omer Gunhan

**Affiliations:** ^1^Department of Oral and Maxillofacial Surgery, Faculty of Dentistry, Ondokuz Mayıs University, 55139 Samsun, Turkey; ^2^Department of Oral Diagnosis and Radiology, Faculty of Dentistry, Ondokuz Mayıs University, 55139 Samsun, Turkey; ^3^Department of Pathology, Gülhane Military Medical Academy, 06020 Ankara, Turkey

## Abstract

Osteomas are benign, slow-growing osteogenic tumors commonly occurring in the craniofacial bones. Osteomas are characterized by the proliferation of compact and/or cancellous bone. It can be of a central, peripheral, or extraskeletal type. The peripheral type arises from the periosteum and is rarely seen in the mandible. The lingual surface and lower border of the body are the most common locations of these lesions. They are usually asymptomatic and can be discovered in routine clinical and radiographic examination. In this paper, we presented a large solitary peripheral osteoma located in the buccal surface of the left posterior mandible and causing facial deformity in a 37-year-old woman. Radiographic examination by computed tomography revealed radiopacity with a well-circumscribed, pedunculated mass approximately 3 cm in size. The osteoma was removed surgically, and no recurrence has been observed.

## 1. Introduction

An osteoma is a benign osteogenic tumor characterized by compact or cancellous bone proliferation. It may be classified as peripheral, central, or extraskeletal. A peripheral osteoma arises from the periosteum, a central osteoma from the endosteum, and an extraskeletal osteoma in the soft tissue [1–4]. The pathogenesis of osteomas is not completely known. They are referred to developmental anomalies, true neoplasms, or reactive lesions triggered by trauma, muscle traction, or infection [1–3, 5]. 

Osteomas are found mainly in the craniofacial bones. A peripheral osteoma (PO) occurs most frequently in the paranasal sinuses. Other locations include the orbital wall, temporal bone, pterygoid processes, and external ear canal [1, 4, 6–8]. As noted in previous reports in the literature, a solitary PO of the jaw bones is quite rare, involving the mandible more often than the maxilla [1, 4, 9]. The most frequent sites affected in the mandible are the posterior body, followed by the condyle, angle, ascending ramus, coronoid process, anterior body, and sigmoid notch [4, 5, 9]. It has been reported that osteomas can occur at any age and that males and females are equally affected [2, 9]. Peripheral osteomas are slow-growing lesions and, clinically, they usually remain asymptomatic. However, when they reach a large size, they can produce swelling and asymmetry.

Patients with osteomas should be evaluated for Gardner's syndrome (GS). This syndrome is an autosomal dominant disease characterized by gastrointestinal polyps, multiple osteomas, skin and soft tissue tumors, and multiple impacted or supernumerary teeth. Intestinal polyps are predominantly adenomas and may progress to malignancy in almost 100% of patients [10, 11]. Because the osteomas may be seen in the earlier stage of GS, the dentists may play an important role in the diagnosis of colonic polyposis [10, 11].

The purpose of this paper is to present a large peripheral osteoma originating from the buccal surface of the mandible and causing asymmetry in a 37-year-old woman.

## 2. Case Report

**Figure 1:**
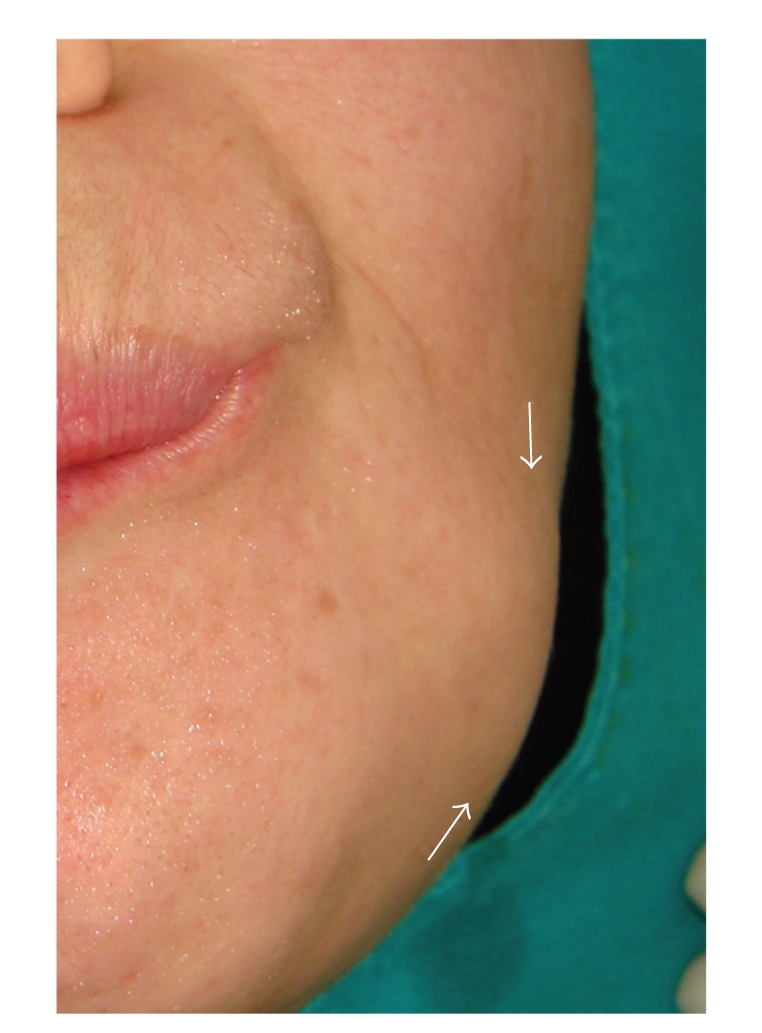
Extraoral photograph shows a swelling on the left posterior body of the mandible.

**Figure 2:**
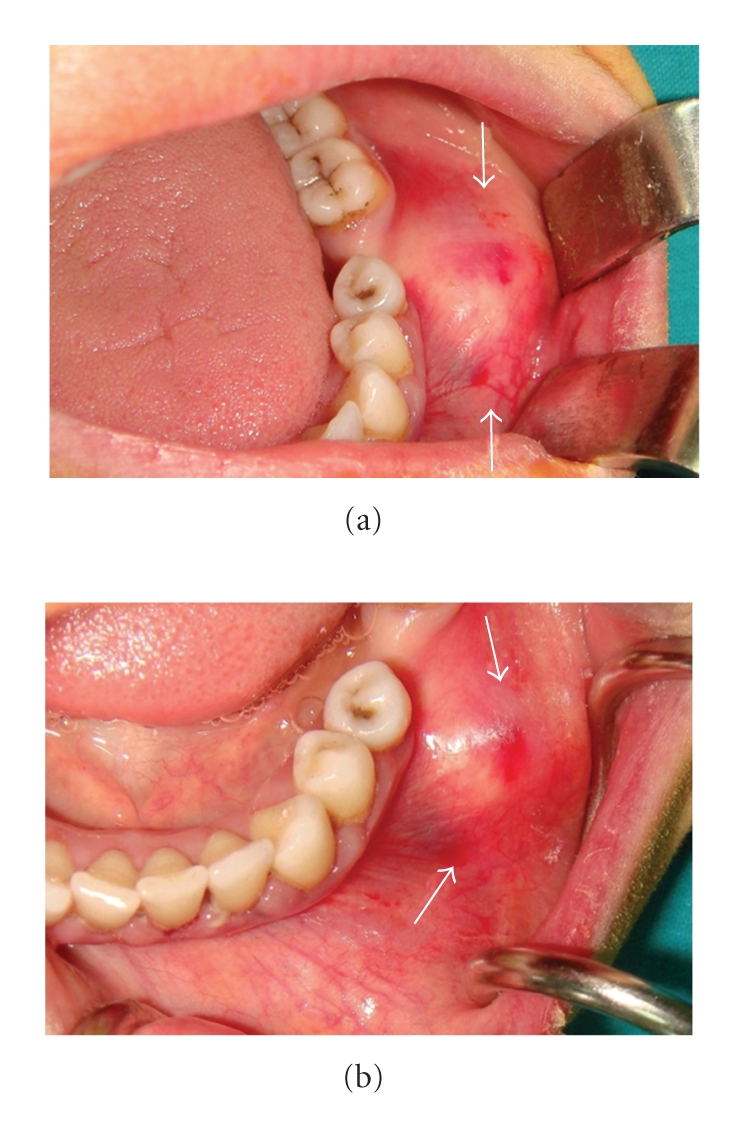
Intraoral view showing a well-defined, round swelling covered by normal oral mucosa on the buccal plate of the left posterior mandible.

**Figure 3:**
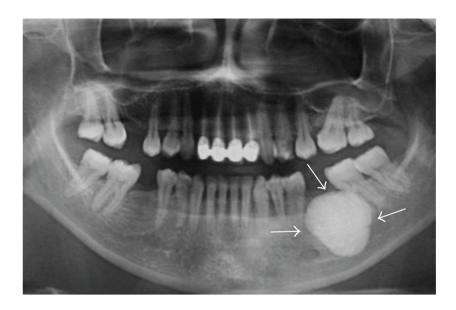
Panoramic radiograph showing a solitary, round, 3 × 3 cm well-defined radio-opaque mass without a radiolucent rim on the left side of the body of the mandible. The lesion extended distally of the second premolar till the mesial aspect of the second molar distal root.

**Figure 4:**
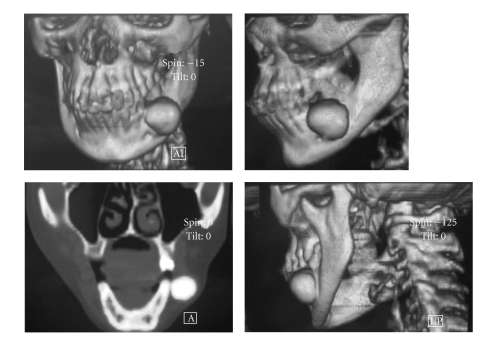
Coronal Computed tomography (CT) showing a large, well-circumscribed, pedunculated mass attached to the buccal surface of the left mandibular body. Three-dimensional reconstruction image showing localisation and extending of the lesion.

**Figure 5:**
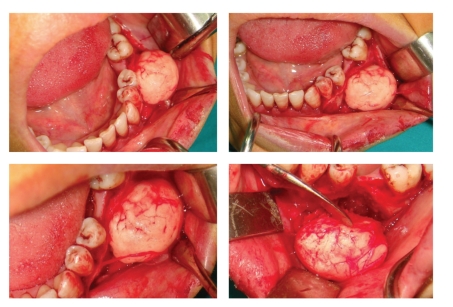
Mucoperiostal flap was removed and the entire lesion was found out.

A 37-year-old woman was referred to the Oral Diagnosis and Radiology Department with a complaint of facial and intraoral swelling on the posterior buccal aspect the left-side mandible. She had been aware of the slow but steady increase in the size of the lesion over the past six years. The lesion was not associated with pain, and there was no problem with mouth-opening or chewing. She had no previous facial trauma, and her medical history was not contributory. Clinical examination revealed extraoral swelling on the left side (Figure 1). The regional lymph nodes were nonpalpable. Intraoral examination revealed a well-defined, round, immobile mass on the buccal plate of the left posterior mandible and buccal expansion. The lesion was bony-hard on palpation. The overlying oral mucosa was normal (Figure 2). There was no pain, tenderness, or paresthesia. The mandibular first molar had been extracted previously. All of the posterior teeth were vital (positive responses to electric pulp testing). A solitary, round, 3 × 3 cm well-defined radio-opaque lesion without a radiolucent rim of mandible was detected with panoramic radiography and computed tomography (CT). The lesion extended distally of the second premolar to the mesial aspect of the second molar distal root (Figures 3 and 4). A CT scan demonstrated a large, pedunculated mass attached to the buccal surface of the left mandibular body. These clinical and radiographic features were sufficiently supportive of the working diagnosis of peripheral osteoma. There were no features of Gardner's syndrome. Because the lesion was actively growing and caused facial swelling, the patient was prepared for surgery. Under local anesthesia, the lesion was totally removed using a chisel and rotary instruments via an intraoral approach, and curettage of the cavity was undertaken (Figure 5). Intraoperatively, the inferior alveolar nerve was determined and preserved. Postoperatively, the patient received systemic antibiotic, analgesic, and mouthwash for 7 days. 

**Figure 6:**
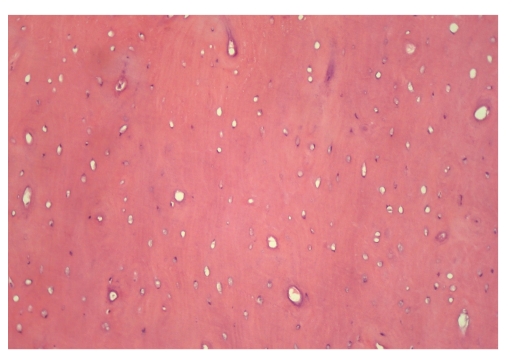
Microscopic features of peripheral osteoma consisting of mature lameller compact bone (haematoxylin-eosin, original magnification ×200).

The patient presented for a postoperative visit and suture removal a week later, and the healing was progressing normally. There were no postoperative complications. The surgical specimen was submitted for histopathological examination. Tissue specimens were fixed in 10% formaldehyde and then decalcified in 8% formic acid solution. They were processed routinely and paraffin embedded. Tissue blocks were cut with 6 *μ* thickness and slides were stained with hematoxylin and eosin. The histopathologic diagnosis confirmed the clinical diagnosis of peripheral osteoma. Microscopic examination of the specimen revealed a hard mass consisting entirely of dense lameller compact bone (Figure 6). The patient was scheduled for regular followup. At the 8-month followup, the area healed well and filled in with bone of normal density. 

## 3. Discussion

Osteomas real prevalence is unknown. Sondergaard et al. [12] in their study demonstrated that the prevalence of osteoma in 50 patients with ulcerative colitis is 4% and 2% in the control group. It has been reported that osteomas have no sex predilection [2, 9]. PO of the jaw bones is quite rare. These lesions are more frequent in the mandible than the maxilla. Sayan et al. [4] reported finding 22.85% of the lesions in the mandible and 14.28% in the maxilla in their study; also, Kaplan et al. [2] reported that 81.3% of cases occurred in the mandible, Chaurasia and Balan [9] reported 83%, and Woldenberg et al. [7] reported 64%.

The lingual surface and lower border of the body are the most common locations of mandibular lesions [5, 7, 13–16]. Rarely, as in our case, the lesions are located on the buccal aspect of the body of the mandible. 

The exact etiology and pathogenesis of peripheral osteoma is unknown. Neoplastic and reactive causes have been suggested as possible etiologic factors. Kaplan et al. [2, 3] and Woldenberg et al. [7] suggested that some peripheral osteomas may be reactive rather than neoplasms, probably associated with trauma. Also, some authors have reported that as many of the peripheral osteomas are located on the lower border of the mandible, it is possible that muscle traction plays a role in the development of peripheral osteomas [5, 7]. However, in the case described in this paper, we have no information as to the possible cause, there being no history of previous trauma or infection.

Clinically, peripheral osteoma appears as an unilateral and well-circumscribed mass ranging from 10 to 40 mm in diameter [7, 9]. Lesions are usually asymptomatic and can be discovered in routine clinical and radiographic examination. Sometimes, depending on the location and size of the lesion, it may cause swelling, facial asymmetry, and functional impairment [5, 7–9, 13, 17, 18]. The swelling is usually painless. In our case, the lesion had reached significantly large dimensions and caused facial asymmetry, without any other clinical symptoms.

Radiographic findings: panoramic radiography or computed tomography is used for imaging; however, as demonstrated in this case, the CT is the best imaging modality for determining the location and real extension of the lesion [7–9, 13, 16, 17, 19]. Peripheral osteomas, in most cases, are easy to recognize because of their classic radiographic findings. On radiological imaging, a peripheral osteoma of the mandible is a classically well-circumscribed, round or oval, mushroom-like radiopaque mass with distinct borders [5, 9, 20, 21]. The lesion may be sessile and attached to the cortical plates with a broad base. If a peripheral osteoma is pedinculated, a narrow contact area can be seen between the lesion and the compact bone. In our case, the lesion consisted of dense, uniformly opaque compact bone with a narrow pedicle demonstrated by CT. 

Differential diagnosis: peripheral osteoma should be differentiated from several pathologic entities, such as exostoses, osteoblastoma, and osteoid osteoma, late-stage central ossifying fibroma, or complex odontoma. Exostoses are bony excrescences that usually stop growing after puberty, differentiating them from osteomas [15, 20]. The borders of central ossifying fibromas are well-defined, and a thin, radiolucent line may separate it from the surrounding bone. A sclerotic border may be present in the bone next to the lesion [22].

Osteoblastomas and osteoid osteomas are more frequently painful and grow more rapidly than peripheral osteomas [4, 5]. A complex odontoma presents as a well-defined radiopacity situated in bone, but with a density that is greater than bone and equal to or greater than that of a tooth. It is also surrounded by a narrow radiolucent rim [22]. 

Removal of an asymptomatic peripheral osteoma is not generally necessary. Surgical intervention is indicated only if it becomes large enough to cause facial asymmetry and functional impairment [1, 7, 9, 17, 18]. Surgical excision is usually simple in pedinculated peripheral osteomas. In the case of mandibular peripheral osteomas, an intraoral approach is preferable to an extraoral approach mainly for cosmetic reasons, as in our case.

## 4. Conclusion

We have presented a case of a large osteoma on the buccal surface of the mandibular body. The lesion had grown slowly for six years and caused intraoral swelling and facial asymmetry. Following histological diagnosis, surgical excision was done. Recurrence of peripheral osteoma after surgical excision is extremely rare. However, it is appropriate to provide both periodic clinical and radiographic followup after surgical excision of a peripheral osteoma.
